# Normative data for accommodative facility and vergence facility in a sample of African school children aged 8–17 years

**DOI:** 10.3389/fnins.2026.1742375

**Published:** 2026-01-26

**Authors:** Charles Darko-Takyi, Ebenezer Manu, Victoria Yirrah, Sandra Owusu, Kumi Owusu Boakye, Carl Halladay Abraham, Kwame Okyere Osei

**Affiliations:** 1 Department of Ophthalmic Science, School of Optometry and Vision Science, University of Cape Coast, Cape Coast, Ghana; 2 Department of Eye, Seventh Day Adventist Hospital, Sunyani-Fiapre, Ghana; 3 Department of Optometry, Malaika Medical Center, Tarkwa, Ghana; 4 Department of Eye, Iran Clinic, Accra, Ghana; 5 Department of Clinical Optometry, School of Optometry and Vision Science, University of Cape Coast, Cape Coast, Ghana

**Keywords:** binocular accommodative facility, Ghanaian children, monocular accommodative facility, normative data, vergence facility

## Abstract

**Background/objectives:**

The existing literature on normative data for accommodative facility (AF) in African populations is limited to high school students. There is no normative data for vergence facility (VF) in African children, so there are no benchmarks for comparison in case analysis, diagnosis, and management. The study aimed to establish normative data for AF in children aged 8–12 years. Additionally, the study sought to determine normative data for VF in children aged 8–17 years in the Cape Coast metropolis, Ghana.

**Methods:**

Normal children (510) were recruited through a comprehensive oculo-visual examination of 2,300 basic school-going children, aged 8–17 years. AF was measured with a ± 2D flipper lens for 1 min. VF was measured with a 3-base-in/12 base-out flipper prism for 1 min. Normative data were derived using the median with interquartile ranges (IQR) and considering the spread of data within the minimum and maximum ranges.

**Results:**

A median value of 13 cpm with IQR of 4 cpm was determined for monocular accommodative facility (MAF). The normative central tendency for MAF for school children 8–17 years ranges from 9 to 17 cpm; data were widely spread, with a minimum of 4 and a maximum of 20 cpm. A median value of 13 cpm with IQR of 3 cpm was determined for the binocular accommodative facility (BAF). The normative central tendency for BAF for school children aged 8–17 years ranged from 9 to 14 cpm; data were widely spread, with a minimum of 5 and a maximum of 20 cpm. A median value of 14 cpm with IQR of 4 cpm was determined for VF. The normative central tendency for VF for school children 8–17 years ranged from 10 to 18 cpm; data were widely spread, with a minimum of 6 and a maximum of 21 cpm.

**Conclusion:**

The normative data apply only to similarly aged Ghanaian children and serve as standards for comparison to clinical data for MAF, BAF, and VF during case analysis.

## 1 Introduction

Accommodative facility (AF) assesses the dynamics of accommodative responses (von Noorden and Campos, 2002; Liu et al., 1979), examines the speed of changes in accommodation (Bertil et al., 2001), and reflects the interaction between accommodation and vergence (Siderov and Johnston, 1990). Vergence facility (VF) testing evaluates the ability of the fusional vergence system to respond quickly and accurately to changing vergence demands over time (Gall et al., 1998). The flexibility of accommodation and vergence—encompassing accommodative and vergence dynamics—is essential for shifting focus from near to distant targets and vice versa during daily activities. Difficulties with these functions place greater strain on the visual system, leading to binocular vision disorders and symptoms such as visual discomfort and asthenopia (Cooper et al., 2011). With heightened academic demands among school children, the stress on the accommodative and vergence systems would rise (Elsiddig A. and Alrasheed H., 2017). Consequently, the occurrences of accommodative and vergence infacilities are likely to rise in the population. Analyzing AF and VF results and comparing them to population-specific normative data is crucial in modern optometric practice.

Interracial, ethnic, and age differences in normative data for binocular vision parameters have been reported (Hussaindeen et al., 2017; Chen and Abidin, 2002; Hussaindeen et al., 2015; Majumber, 2015). These differences are evident in studies on AF among school children (Scheiman et al., 1988; Chen and Abidin, 2002; Gierow et al., 2014), teenagers (Wajuihian, 2019; Darko-Takyi et al., 2022), university students (Chikuse et al., 2022), and young adults (Alrasheed et al., 2024). The difference is again evident in studies on VF among school children (Chen and Abidin, 2002; Gierow et al., 2014) and young adults (Gall et al., 1998; Momeni-Moghaddam et al., 2014). Among children, the reference values for monocular accommodative facility (MAF) and binocular accommodative facility (BAF) range from 5 to 26 cpm and 2 to 26 cpm, respectively (Scheiman et al., 1988; Gierow et al., 2014; Chen and Abidin, 2002). Among teenagers, the reference values for MAF and BAF range from 6 to 13 cpm and 5 to 12 cpm, respectively (Wajuihian, 2019; Darko-Takyi et al., 2022). In adults, the reference values for MAF and BAF range from 5 to 10 cpm and 4 to 12 cpm, respectively (Alrasheed et al., 2024; Chikuse et al., 2022). The reference values for VF for children range from 5 to 26 cpm (Chen and Abidin, 2002; Gierow et al., 2014; Gall et al., 1998). Among adults, the reference values for VF range from 10 to 19 cpm (Chen and Abidin, 2002; Gall et al., 1998; Momeni-Moghaddam et al., 2014). Variations in these results are attributed to the ocular anatomical differences among populations (Wang et al., 2012; Blake et al., 2003), which influence refractive (Kleinstein et al., 2003; Dadeya et al., 2001) and accommodative states (Jimenez et al., 2004; Chen and Abidin, 2002). The standards reported, besides being population-specific, are also limited by the discrepancies in the techniques, targets, and measurement protocols used for testing AF and VF across populations.

Optometrists in Africa mostly rely on the standards established for American populations (Scheiman and Wick, 2014) when interpreting and analyzing AF and VF results, as well as monitoring treatment. This is due to the lack of available age-appropriate, population-specific normative data for these measures. Such practice can cause inaccuracies in diagnosing and managing non-strabismic binocular vision anomalies among African populations. Acquiring age-specific normative data for AF and VF tailored to the African population is crucial for accurate diagnosis and effective management of these conditions (Hussaindeen et al., 2017; Hussaindeen et al., 2015; Chen and Abidin, 2002; Majumber, 2015). In Africa, Wajuihian (2019) and Darko-Takyi et al. (2022) reported normative data for AF among teenagers, whereas Chikuse et al. (2022) provided data for university students. The AF data from these studies differ from those of younger African children, as Hussaindeen et al. (2017) found a significant increase in mean values between younger children and teenagers in India. Additionally, Scheiman et al. (1988) observed lower mean AF measures in school children compared to adults. Moreover, there is no reported normative data on VF for any African population. This study aims to establish normative data for AF in children aged 8–12 years in Ghana and for VF among children aged 8–17 years in Ghana.

## 2 Materials and methods

### 2.1 Ethical considerations

This study conformed to the Code of Ethics of the World Medical Association (Declaration of Helsinki). The study was ethically approved by the University of Cape Coast Institutional Review Board (Ref: UCCIRB/CHAS/2019/173). The Cape Coast Metro Education Directorate, Ghana, and the head teachers of the sampled schools granted permission. Parents and guardians gave written informed consent, and school children gave their assent to participate in the study.

### 2.2 Study design and sampling

A cross-sectional study using a multistage sampling technique was conducted among primary school children in the Cape Coast metropolis, Ghana. The minimum sample size was calculated using the formula for normative data [(Z1-a/2)^2^SD^2^]/d^2^. Z1-a/2 represents the standard normal variate at a 95% confidence interval (*p* < 0.05), which is 1.96. SD refers to the standard deviation of normative quantitative variables, with 2.50 cpm for accommodative facility and 3 cpm for vergence facility. “d” is taken as 0.5 cpm, representing the allowable error or precision in estimating the normative data for accommodative and vergence facilities. Considering a design effect of three and accounting for a 10% attrition rate, the minimum sample sizes calculated for the normative study of AF and VF were 317 and 456, respectively.

The schools in the Cape Coast metropolis were clustered into six according to their location. Simple random sampling was employed to select two schools from each of the six clusters. At each of the 12 selected schools, a minimum of 45 normal participants were randomly selected.

### 2.3 Data collection procedure

#### 2.3.1 Questionnaire administration

The revised Convergence Insufficiency Symptom Survey (CISS) questionnaire (Borsting et al., 2003) (a valid and reliable 15-symptom tool to distinguish between patients with normal binocular vision and those with convergence insufficiency or other binocular vision issues) was administered to eliminate participants with symptoms of non-strabismic binocular vision anomaly, as studies indicate symptoms significantly overlap (Davis et al., 2016; Marran et al., 2006). Each question was read verbatim, and subjects were asked to rate the frequency of their symptoms on a scale of 0–4 (0 indicates never; 1, infrequently; 2, sometimes; 3, fairly often; and 4, all of the time).

#### 2.3.2 Oculo-visual screening phase

The Bailey Lovie LogMAR chart and N-notation charts were used to assess distance and near visual acuity, respectively. Stereoacuity and suppression were evaluated with the TNO stereoscopic chart. Unilateral cover testing with prism bar neutralization was performed using an occluder and prism bar. External and internal ocular examinations were conducted with a handheld slit lamp and a direct Keeler ophthalmoscope, respectively. Non-cycloplegic objective refraction was performed using the streak retinoscope; subjective refraction (using the forging technique) was carried out with the trial lens set.

#### 2.3.3 Exclusion criteria

Participants with a CISS score greater than 16 (Rouse et al., 2009), best corrected visual acuity in one or both eyes worse than 0.0 LogMar at distance or at near, stereoacuity greater than 60 s of arc, ocular suppression, constant or intermittent strabismus, nystagmus, and ocular disease were excluded from the study.

#### 2.3.4 Accommodative facility testing

AF was assessed using ±2 D flipper lenses while focusing at near (40 cm) on N6 black reading letters on a white background. The child first focused the target through the +2D side of the flipper and reported that the letters became clearer. The examiner quickly flipped to the -2D side as the child continued to focus, and the child reported that it became clear again. The test lasted 60 s; a cycle was defined as the ability to clear both the plus and minus sides of the ±2D flipper lenses. The number of cycles within 1 min of testing was recorded as cycles per minute (cpm). The test was performed monocularly (right and left eyes) and binocularly, with results recorded accordingly. The order of testing was randomized for the right eye, left eye, and both eyes. The participants were made to rest for one minute between each test for consistency in data collection, as there is no specified official break time in standard clinical practice. Before testing for AF, the children were asked to read the N6 letters as confirmation of reading fluency. The choice of N6 optotypes for all participants was to ensure that the same stimulus was used for all participants during testing to avoid a systematic error or bias.

#### 2.3.5 Vergence facility testing

VF was measured for 1 min using the 3BI/12BO flipper prism, focusing at 40 cm on black N6 letters on a white background. The 3 base-in/12 base-out flipper prism was alternately flipped in front of both eyes, and the child reported seeing a single target when it appeared as one. The base-in prism was introduced first, followed by the base-out prism. If the 12-base-out prism was introduced first, the induced vergence adaptations from the convergence responses could temporarily bias subsequent base-in measurements (Sassonov et al., 2010). One cycle was defined as the ability to see the target as single through the 3 base-in and 12 base-out prisms alternately for both eyes. The number of cpm was recorded.

### 2.4 Data analysis

Data were analyzed using IBM SPSS version 23. Boxplots and quantile-quantile plots were used to identify outliers; all outliers were replaced with the median. The Kolmogorov–Smirnov test was used to test for normality. Descriptive analysis was done using the medians, interquartile ranges (IQR), and percentiles; the means with standard deviation and their 95% confidence intervals were also presented. A Mann–Whitney U test was used to determine the differences in AF and VF among gender and age categories. The Wilcoxon signed-rank test was used to determine differences in AF between the right and left eyes. A Spearman rho correlation was used to determine the association between age, and AF and VF. A *p*-value of ≤ 0.05 was defined as statistically significant. A clinically significant difference was defined as a median difference within the IQR (within the central tendency); this difference cannot push the data outside the range of minimum and maximum to cause potential asthenopia.

## 3 Results

The number of participants enrolled in the study were 2,300 of which 1,624 (70.60%) were excluded [symptomatic (906), best corrected visual acuity worse than 0.0 logMAR at distance and at near (299), ocular diseases (161), strabismus (147), stereoacuity less than 60 arcsec (63), suppression (37), and nystagmus (14)] and 166 (7.2%) were dropped out (non-complaince with AF and VF test instructions). The sample excluded were referred to the University of Cape Coast Eye Clinic for treatment. The final normal sample was 510, of which 263 (51.57%) were males, and 247 (48.43%) were females. The final sample age (ranged: 8–17 years; mean: 12.37 ± 2.18 years) was not normally distributed (*p* = 0.0001). The quantifiable screening parameters for the normal sample (510) are stereoacuity (mean ±1SD = 57.26 ± 9.31 and median = 60.00 IQR 0), distance visual acuity (mean ±1SD = −0.02 ± 0.078; median = −0.100 IQR 0.1), and CISS score (mean ±1SD = 8.45 ± 4.640; median = 9 IQR 7).

**Figure 1:**
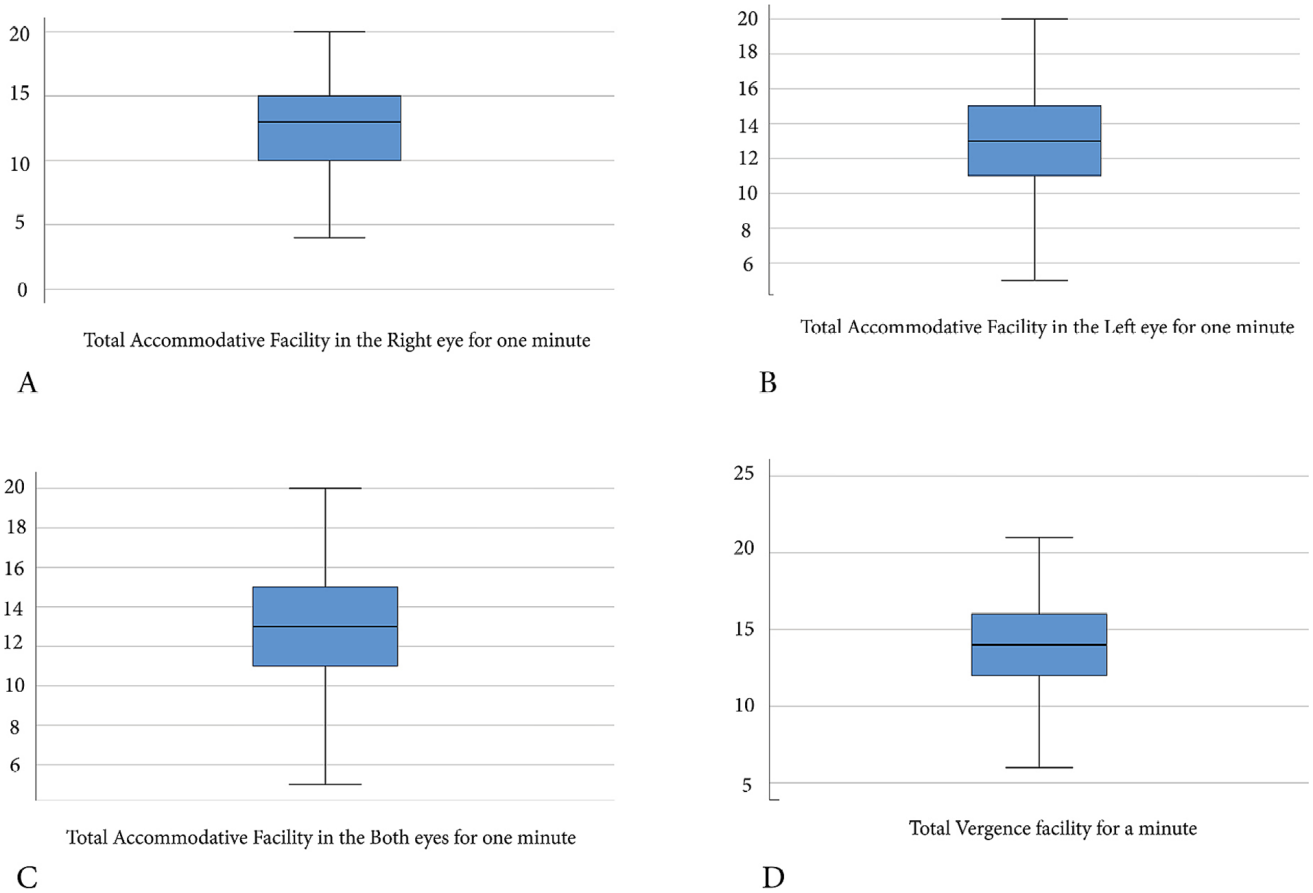
Boxplot indicating range of normative data for accommodative and vergence facilities for a sample of school children in Cape Coast, Ghana. **(A)** Median (interquartile range) for monocular accommodative facility (right eye) is 13 (5) cpm, the spread of normative range with minimum and maximum normative data point of 4–20 cpm. **(B)** Median (interquartile range) for monocular accommodative facility (left eye) is 13 (4) cpm, with a wide spread normative range, minimum and maximum data points of 4–20 cpm. **(C)** Median (interquartile range) for binocular accommodative facility is 13 (3) cpm, with wide spread normative range, minimum and maximum data point of 5–20 cpm. **(D)** Median (interquartile range) for vergence facility is 14 (4) cpm, with widespread normative range, minimum and maximum data points of 6–21 cpm.

AF and VF measures for the normal participants were not normally distributed (Tables 1, 2). The normative data thus presented (Table 1; Figure 1) describes not just the means, standard deviation, and 95% confidence intervals, but also the median, interquartile ranges, and percentiles. There was a significant difference in MAF between right (mean rank: 186.88) and left (mean rank: 208.57) eyes (*Z* = 3.085, *p* = 0.002), thus, both measures were presented (Tables 1, 2).

**Table 1:** Description of normative data for accommodative facility and vergence facility for school children in Cape Coast, Ghana.

Measures	MAF (RE) Cpm	MAF (LE) Cpm	BAF cpm	VF Cpm
Mean ± SD	12.49 ± 3.44	12.73 ± 3.14	12.35 ± 2.37	13.47 ± 2.80
95% CI	12.19–12.79	12.46–13.01	12.14–12.55	13.23–13.72
25th percentile	10.00	11.00	11.00	12.00
Median	13.00	13.00	13.00	14.00
IQR	5.00	4.00	3.00	4.00
75th percentile	15.00	15.00	14.00	16.00
95th percentile	18.00	18.00	16.00	18.00
KS *p*-value	0.0001	0.0001	0.0001	0.0001

MAF, monocular accommodative facility; BAF, binocular accommodative facility; VF, vergence facility; RE, right eye; LE, left eye; CI, confidence interval; IQR, interquartile range; cpm, cycles per minute; Kolmogorov–Smirnov (*p*-value less than 0.05 indicates the data is not normally distributed).

**Table 2:** Distribution of parameters of accommodative facility and vergence facility among gender and age category for school children in Cape Coast, Ghana.

Parameters	Gender	Mean ± SD	Percentiles				
			25th	Median	IQR	75th	90th
MAF (RE)	Male	12.88 ± 3.38	11.00	13.00	4	15.00	17.00
	Female	12.07 ± 3.45	10.00	12.00	5	15.00	16.00
MAF (LE)	Male	13.09 ± 3.00	12.00	13.00	3	15.00	16.00
	Female	12.36 ± 3.35	10.00	13.00	5	15.00	16.00
BAF	Male	12.47 ± 2.43	11.00	13.00	4	14.00	16.00
	Female	12.21 ± 2.30	11.00	12.00	3	14.00	16.00
VF	Male	13.67 ± 2.98	12.00	14.00	4	16.00	17.00
	Female	13.26 ± 2.57	12.00	14.00	3	15.00	16.00
	Age category						
MAF (RE)	Young children	12.72 ± 3.58	10.50	13.00	5	15.00	18.00
	Teeenage	12.25 ± 3.28	10.00	12.00	4	14.00	18.00
MAF (LE)	Young children	12.93 ± 3.10	12.00	13.00	3	15.00	18.00
	Teenage	12.53 ± 3.17	11.00	13.00	4	15.00	18.00
BAF	Young children	12.48 ± 2.43	11.00	13.00	3	14.00	16.00
	Teenage	12.21 ± 2.30	11.00	12.00	3	14.00	16.00
VF	Young children	13.85 ± 2.81	12.00	14.00	4	16.00	18.00
	Teenagee	13.09 ± 2.73	11.00	13.00	4	15.00	17.30

MAF, monocular accommodative facility; BAF, binocular accommodative facility; VF, vergence facility; RE, right eye; LE, left eye.

Mean ranks for males were significantly greater than for females (Table 3) for MAF and VF. The observed median differences of 1 cpm or less, however, for MAF and VF are not clinically meaningful. The mean ranks of the younger children (8–12 years) were greater than those for the teenage children (13–17 years) for the AF and VF (Table 3). This difference was, however, statistically significant for VF only (Table 3). The median difference in VF of 1 cpm between young children and teenagers was not clinically meaningful (Table 2).

**Table 3:** Comparison of accommodative and vergence facility among gender and age categories for school children in Cape Coast, Ghana, in Mann–Whitney U test.

Parameter	Gender	Mean rank	Mann–Whitney U	*Z*-value	*P*-value
MAF (RE)	Male	272.04	28,130	−2.263	*0.009
	Female	237.89			
MAF (LE)	Male	271.39	28,302	−2.528	*0.011
	Female	238.58			
BAF	Male	264.84	30023.5	−1.477	0.140
	Female	245.55			
VF	Male	266.86	29,493	−1.809	*0.07
	Female	243.4			
	Age category				
MAF (RE)	Young children	267.57	29409.50	−1.874	0.061
	Teenage	243.24			
MAF (LE)	Young children	265.53	29933.00	−1.559	0.119
	Teenage	245.31			
BAF	Young children	264.22	30270.5	−1.357	0.175
	Teenage	246.65			
VF	Young children	278.04	26716.5	−3.507	*0.0001
	Teenage	232.60			

MAF, monocular accommodative facility; BAF, binocular accommodative facility; VF, vergence facility; RE, right eye; LE, left eye. * Means statistically significant.

**Figure 2:**
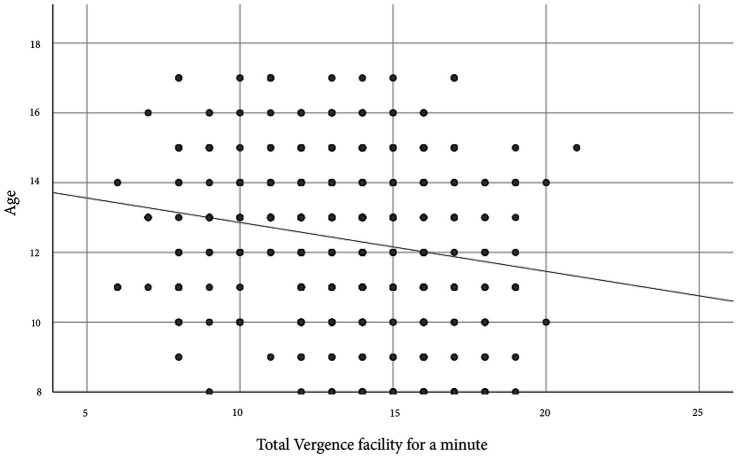
Scatterplot indicating a significantly weak negative Spearman rho correlation between age in years and vergence facility in cycles per minute (cpm) for a sample of school children in Cape Coast, Ghana. The correlation coefficient, r_s_ = −0.179, and the *p* < 0.0001.

There was a very weakly significant negative correlation between age and VF (Figure 2). There was no significant correlation between age and MAF for the right eye (r_s_ = −0.078, *p* = 0.077), left eye (r_s_ = −0.076, *p* = 0.086), and binocular accommodative facility (BAF) (r_s_ = −0.04, *p* = 0.369). There were moderately significant positive correlations between VF and MAF [right eye (r_s_ = 0.631, *p* = 0.0001), left eye (r_s_ = 0.633, *p* = 0.0001)] and BAF (r_s_ = 0.580, *p* = 0.0001).

## 4 Discussion

### 4.1 Intepretation of normative data

As the data were not normally distributed, the main reference descriptive guidelines for interpretation are the median with the interquartile ranges, and the spread of the data considering the minimum and maximum values. The mean data with standard deviations, however, can also guide practitioners. Considering the median with interquartile range (Table 1) and the mean with standard deviation (Table 1), the range of standards for MAF among school children in Cape Coast, Ghana, is 9 to 17 cpm. The median with interquartile range for the BAF (Table 1), along with its mean and standard deviation (Table 1), indicates that the standards for BAF in the population range from 9 to 14 cpm. The median with interquartile range (Table 1) and the mean with standard deviation (Table 1) indicate that the standards for VF among Cape Coast school children range from 10 to 18 cpm. These ranges described above represent the central tendencies for the normative data. Considering the widely spread nature of the normative data points (minimum and maximum values), the data should be interpreted as normal, with emphasis on the absence of binocular vision-related symptoms. The range of standards for BAF was lower than MAF; also, the range of standards for VF was greater than that for the accommodative facility. In comparing the standard data (Table 1) with patients’ clinical data for Ghanaian children 8–17 years old, any measure lower than this range can be interpreted as low, and that above this range may be interpreted as exaggerated. Even though the study found statistically significant median differences between the standards for males and females, the observed median differences of 1 cpm for MAF and BAF are not clinically meaningful, as the values fall within their interquartile ranges. Also, the observed differences in VF between young children and teenage children are not clinically meaningful. The normative data described (Table 1) applies to both genders and ages (8–17 years), as any observed differences are not clinically meaningful. During clinical case analysis for Ghanaian children within the age of 8–17 years, practitioners should compare patients’ data to the standards described (Table 1) for the population, instead of the gender based standards (Table 2). The normative data for the VF determined (Table 1) is novel for an African population. The normative data derived for AF for Ghanaian children below the age of 12 years are novel, as a previous study by Darko-Takyi et al. (2022) included older-aged children up to 17 years old.

### 4.2 Comparing accommodative facility and vergence facility normative data with the literature

Tables 4, 5 compare the results of the present study to standards in other populations. The MAF result was comparable to Burge (1979), regardless of differences in population, sample size, and age ranges (Table 4). The AF results in the present study are higher compared to those among teenage children in the Central region of Ghana (Darko-Takyi et al., 2022) and in South Africa (Wajuihian, 2019), but lower compared to those in India (Hussaindeen et al., 2017). For children below the age of 12 years (Table 4), the range of AF standards in the present study is comparable to that among Indians (Hussaindeen et al., 2017), higher compared to that in Spanish children (Jimenez et al., 2004), standards by Scheiman and Wick (2014) and Swedish children (Gierow et al., 2014), and lower compared to standards in Malaysian children (Chen and Abidin, 2002).

**Table 4:** Comparing normative data for accommodative facility for school children in Cape Coast, Ghana, with standards in other populations.

Study	Country	Sample size	Flipper device and target size	Age range/years	MAF/cpm	BAF/cpm
Present study	Ghana	510	± 2D@ 40 cm	8–12 13–17	RE:12.72 ± 3.58 LE:12.93 ± 3.10 RE:12.25 ± 3.28 LE:12.53 ± 3.17	12.48 ± 2.43 12.21 ± 2.30
Darko-Takyi et al. (2022)	Ghana	1,261	± 2D @ 40 cm	11–17	9.80 ± 3.20	9.40 ± 3.30
Chen and Abidin (2002)	Malaysia	60	± 2D @ 40 cm	6 to12	20.08 ± 6.43	19.77 ± 6.26
Jimenez et al. (2004)	Spain	-	-	6 to 12	7.16 ± 3.24	3.84 ± 2.91
Scheiman and Wick (2014)	USA	–	± 2D @ 40 cm	6 7 8–12 13–30	5.50 ± 2.50 6.50 ± 2.00 7.00 ± 2.50 11.00 ± 5.00	3.00 ± 2.50 3.50 ± 2.50 5.00 ± 2.50 10.00 ± 5.00
Gierow et al. (2014)	Sweden	37	± 2D @ 40 cm	8–13	6.70 ± 4.40	5.50 ± 3.90
Hussaindeen et al. (2017)	India	936	± 2D @ 40 cm	7–12 13–17	11.00 ± 4.00 14.00 ± 5.00	10.00 ± 4.00 14.00 ± 5.00
Wajuihian (2019)	South Africa	1,211	± 2D @ 40 cm	13–18	8.70 ± 3.40	8.80 ± 3.50
Burge (1979)	Germany	30	± 2D @ 40 cm	6–30	RE:12.60 ± 4.60 LE:11.60 ± 4.25	7.05 ± 4.25

MAF, monocular accommodative facility; BAF, binocular accommodative facility; RE, right eye; LE, Left eye.

**Table 5:** Comparing normative data for vergence facility for school children in Cape Coast, Ghana, with standards in other populations.

Author (year)	Country	Sample size	Device and test distance	Age range/years	Vergence facility/cpm
Present study	Ghana	510	3BI/12BO at 40 cm	8–12 13–17	13.85 ± 2.81 13.09 ± 2.73
Scheiman and Wick (2014)	USA	–	3BI/12BO at 40 cm	6 to 30	15.00 ± 3.0
Gierow et al. (2014)	Sweden	37	3BI/12BO at 40 cm	8–13	9.30 ± 3.8
Hussaindeen et al. (2017)	India	936	3BI/12BO at 40 cm	7–12 13–17	12.00 ± 4.0 14.00 ± 4.0
Chen and Abidin (2002)	Malaysia	60	3BI/12BO at 40 cm	6 to 12	20.18 ± 5.0
Gall et al. (1998)	Houston University students, either race	20	3BI/12BO at 40 cm	18–25	15 or 16.0 ± 2.6

BI, base-in; BO, base-out.

Despite the difference in sample sizes, the VF for participants in the present study (Table 5) was comparable with that in the study by Hussaindeen et al. (2017) among Indian children of a similar age range. For children under the age of 12 years, VF standards were higher compared to those among Swedish children (Gierow et al., 2014) and lower compared to those among Malaysian children (Chen and Abidin, 2002). The mean of VF in the present study (Table 1) is lower compared to 15.00 ± 3.0 cpm reported by Scheiman and Wick (2014) in a wider age-ranged population, representing a difference of approximately 2 cpm (Table 5). The difference in MAF between the right and left eyes in the present study aligns with other studies (Scheiman et al., 1988; Darko-Takyi et al., 2022; Chen and Abidin, 2002). Accommodative function is monocular (von Noorden and Campos, 2002), and the dominant eye is found to exhibit higher AF than the non-dominant eye (Momeni-Moghaddam et al., 2014; Odigie et al., 2019). The lower BAF measurements compared to the MAF in this study align with previous studies (Scheiman et al., 1988; Burge, 1979; Chen and Abidin, 2002; Jimenez et al., 2004; Scheiman and Wick, 2014; Gierow et al., 2014; Hussaindeen et al., 2017; Darko-Takyi et al., 2022; Wajuihian, 2019). Binocular vision processing involves additional functions and factors such as vergence and fusion, beyond accommodation for each eye, which can interfere with the speed and slow down binocular accommodative function compared to monocular accommodative functions (Kędzia et al., 1999). Moreover, convergence accommodation induced by binocular convergence can further slowdown the binocular accommodative facility. The higher range of VF measures compared to AF measures is comparable to studies by Gierow et al. (2014) and Chen and Abidin (2002).

### 4.3 Comparing AF and VF with demographic parameters

The gender differences in AF and VF in this study were not clinically significant and aligned with findings from a study on Swedish children (Gierow et al., 2014). The absence of a significant link between age and AF matches results from another study on Ghanaian children of a similar age (Darko-Takyi et al., 2022). The inverse relationship between age and VF observed here is similar to findings from a study on Indian children (Hussaindeen et al., 2017). The positive correlation between AF and VF agrees with the study among Indian children (Hussaindeen et al., 2017) and that of Scheiman and Wick (2014).

### 4.4 Limitation

The non-cycloplegic refraction utilized may imply that the children’s refractive system may not have been completely relaxed before VF and AF testing. This may overestimate the accommodative parameters, especially for children with latent hyperopia. However, the forging technique adopted for subjective refraction may correct for this. As the dominant eye exhibits more accommodation than the non-dominant eye, another limitation of the study was the lack of ocular dominance testing. The median imputation of the outliers instead of elimination may have affected the distribution of the data. Again, the lack of the use of standard and measurable methods of assessing reading proficiency may have influenced participants’ responses. However, all children who could not read the target appropriately were dropped out of the study.

## 5 Conclusion

The normative data for MAF and BAF for school children 8–17 years lie within a central tendency of 9–17 cpm and 9–14 cpm, respectively. The data was widely spread, with a minimum of 4 and a maximum of 20 cpm for MAF, and a minimum of 5 and a maximum of 20 cpm for BAF. The normative data for VF for school children 8–17 years lie within a central tendency of 10 to 18 cpm; data is, however, widely spread, with a minimum of 6 and a maximum of 21 cpm. These values serve as standards for comparison to optometric clinical data on AF and VF during binocular vision case analysis for Ghanaian school children of similar ages.

## Data Availability

The raw data supporting the conclusions of this article will be made available by the authors, without undue reservation.
